# Changes in Cytokine Levels and NK Cell Activation Associated with Influenza

**DOI:** 10.1371/journal.pone.0025060

**Published:** 2011-09-23

**Authors:** Stephanie Jost, Heloise Quillay, Jeff Reardon, Eric Peterson, Rachel P. Simmons, Blair A. Parry, Nancy N. P. Bryant, William D. Binder, Marcus Altfeld

**Affiliations:** 1 Ragon Institute of Massachusetts General Hospital, Massachusetts Institute of Technology and Harvard, Massachusetts General Hospital, Harvard Medical School, Boston, Massachusetts, United States of America; 2 Department of Emergency Medicine, Massachusetts General Hospital, Boston, Massachusetts, United States of America; 3 Massachusetts General Hospital Medical Walk-In Clinic, Massachusetts General Hospital, Boston, Massachusetts, United States of America; University of California San Francisco, United States of America

## Abstract

Several studies have highlighted the important role played by murine natural killer (NK) cells in the control of influenza infection. However, human NK cell responses in acute influenza infection, including infection with the 2009 pandemic H1N1 influenza virus, are poorly documented. Here, we examined changes in NK cell phenotype and function and plasma cytokine levels associated with influenza infection and vaccination. We show that absolute numbers of peripheral blood NK cells, and particularly those of CD56^bright^ NK cells, decreased upon acute influenza infection while this NK cell subset expanded following intramuscular influenza vaccination. NK cells exposed to influenza antigens were activated, with higher proportions of NK cells expressing CD69 in study subjects infected with seasonal influenza strains. Vaccination led to increased levels of CD25+ NK cells, and notably CD56^bright^ CD25+ NK cells, whereas decreased amounts of this subset were present in the peripheral blood of influenza infected individuals, and predominantly in study subjects infected with the 2009 pandemic H1N1 influenza virus. Finally, acute influenza infection was associated with low plasma concentrations of inflammatory cytokines, including IFN-γ, MIP-1β, IL-2 and IL-15, and high levels of the anti-inflammatory cytokines IL-10 and IL-1ra. Altogether, these data suggest a role for the CD56^bright^ NK cell subset in the response to influenza, potentially involving their recruitment to infected tissues and a local production and/or uptake of inflammatory cytokines.

## Introduction

Seasonal influenza epidemics result in an estimated 250,000–500,000 deaths per year worldwide [1] and in an average of 200,000 hospitalizations [2] and 36,000 deaths per year in the United States alone [3], [4]. Seasonal epidemics are instigated by the quick spread of influenza virus variants generated by antigenic drift, a process that facilitates the escape of the virus from the host immune system from one outbreak to another. As a result, influenza vaccines need to be reformulated before each annual epidemic, and while yearly immunization has been the most efficient way to prevent influenza so far, vaccines do not provide complete protection [5].

Viral strains included in the vaccine are selected according to surveillance-based forecasts and contains components of influenza B, influenza A H1N1 and influenza A H3N2 viruses, which have accounted for the majority of reported infections for the last three to four decades. In April 2009, a new strain of influenza A (2009 pandemic H1N1) genetically related to swine influenza viruses but capable of efficient human-to-human transmission emerged in Mexico and California and caused the first influenza pandemic since 1968 [6], [7]. While the 2009 pandemic H1N1 virus does not generally cause a more severe illness than the seasonal strains, it can trigger acute pulmonary symptoms due to its ability to infect cells of the lower respiratory tract [8]. Importantly, reassortment between the seasonal strains and the 2009 H1N1 influenza virus could lead to the emergence of variants with increased pathogenicity and/or resistance to current antiviral treatments. Therefore, it is essential to further understand the mechanisms underlying the establishment and the maintenance of an effective immune response during influenza infection.

Recovery from acute influenza infection and resistance to re-infection rely both on the production of neutralizing antibodies targeting the hemagglutinin and the neuraminidase glycoproteins, and on the killing of infected cells by influenza-specific cytotoxic CD8+ T lymphocytes that mostly target conserved viral proteins [9], [10]. In addition, several studies have highlighted the early and pivotal role of innate effector cells, and particularly natural killer (NK) cells, in the control of influenza infection [11]–[27]. Before the onset of the adaptive immune response, NK cells are not only responsible for the production of antiviral cytokines, but they are also directly involved in the rapid elimination of virally infected cells. Moreover, besides their direct antiviral activity, NK cells secrete large quantities of pro- or anti-inflammatory cytokines that directly modulate the quality of the adaptive immune response, mainly via their interaction with dendritic cells [28]. NK cells initially represent a substantial portion of the lymphocyte population that resides in the healthy lungs and are further recruited to the respiratory tract within days of influenza infection [16], [25], [29], [30]. Furthermore, several studies in mice have illustrated that NK cell depletion leads to augmented morbidity and mortality from infection [26], [27], [31] and that impaired activity of NK cells early in the infection results in a delayed clearance of the virus from the lungs [21], [32]. Finally, in humans, severe and/or lethal influenza infection with the 2009 pandemic H1N1 virus has been associated with reduced proportions of NK cells in the peripheral blood and the lungs [13], yet very little is known about changes in the phenotype and function of NK cells in acute 2009 H1N1 influenza infection.

Because of their previously recognized implication in the immune response to influenza, we investigated the phenotype and function of NK cells as well as the cytokine levels in patients with acute influenza infection, comparing infection with 2009 circulating strains of human origin and infection with 2009 pandemic H1N1 influenza virus, or in subjects receiving intramuscular influenza vaccination. We show that acute influenza infection is associated with a depletion of peripheral blood NK cells, and particularly CD56^bright^ NK cells, consistent with their homing to infected tissues and/or with an increased NK cell death. Influenza vaccination and infection both lead to NK cell activation, with the highest levels found in acute influenza with human viruses. Finally, we describe significant changes in cytokine concentrations in the plasma of infected subjects, and notably a marked increase in anti-inflammatory cytokines. These studies provide insights into innate immune responses following influenza antigens exposure in humans.

## Results

### Marked changes in CD56^bright^ NK cell numbers in acute influenza infection and following influenza vaccination

**Figure 1:**
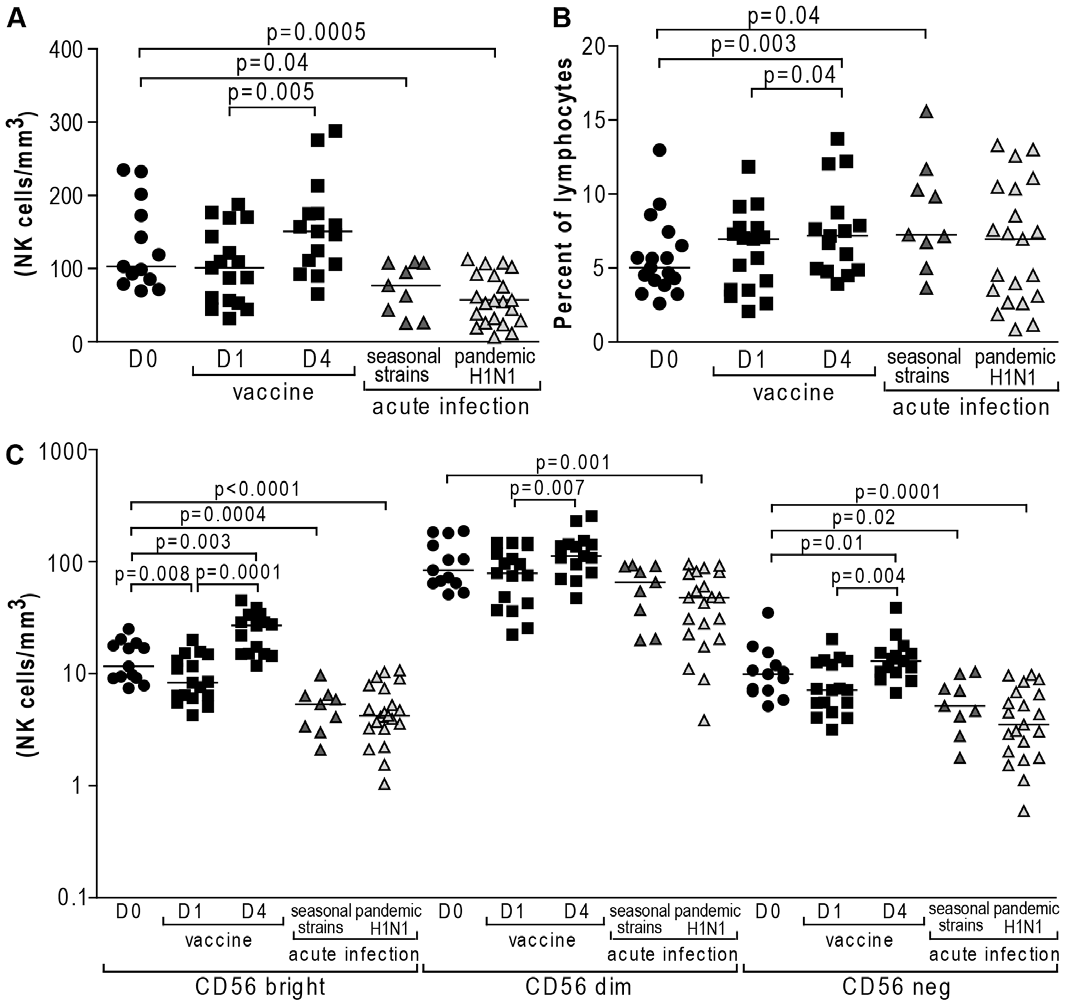
Changes in NK cell numbers and subset distribution in acute influenza infection and influenza vaccination. Dot plots represent (A), absolute numbers of NK cells (B), percentages of total NK cells and (C), absolute numbers of CD56^bright^ (CD3-CD56+CD16−), CD56^dim^ (CD3-CD56+CD16+) and CD56^neg^ (CD3-CD56−CD16+) NK cells from 17 subjects prior to influenza vaccination, and at day 1 and 4 post-vaccination, 9 patients with acute seasonal influenza and 21 with acute 2009 pandemic H1N1 influenza virus infection. Complete blood counts at baseline were only available for 13 of the vaccinated subjects. Horizontal lines indicate the median percentages. Differences where p<0.05 are indicated. (•, healthy subjects prior vaccination; ▪, healthy subjects post-vaccination; dark grey ▴, acutely infected with 2009 seasonal influenza strains; light grey ▴, acutely infected with 2009 pandemic H1N1 influenza virus). D: day post-vaccination.

Expansion of NK cells has been previously described in various human acute viral infections and might represent a crucial step to achieve early effective control of viral replication [33]–[35]. However, whether infection with seasonal and 2009 pandemic H1N1 influenza strains differently affect NK cell expansion and activation has never been investigated. We first examined NK cell numbers in blood samples from study subjects with acute influenza caused by either seasonal or 2009 pandemic H1N1 influenza viruses, or following seasonal intramuscular influenza vaccination. Absolute numbers of NK cells were decreased in patients infected with seasonal (p = 0.04) and 2009 pandemic H1N1 (p = 0.0005) influenza viruses compared to healthy unvaccinated individuals (Fig. 1A), whereas percentages of NK cells relative to the total lymphocyte population were slightly increased in influenza infected patients, notably in seasonal influenza infection (p = 0.04) (Fig. 1B). Of note, diminished NK cell numbers reflected a general lymphopenia in the majority of influenza infected subjects (data not shown).

NK cells are defined as lymphocytes that are CD3-negative and can be further subdivided according to their expression of CD56 and/or CD16 into three subpopulations with distinct phenotypic and functional properties, namely CD56^bright^ (CD3-CD56+CD16−), CD56^dim^ (CD3-CD56+CD16+) and CD56^neg^ (CD3-CD56−CD16+) NK cells [36]–[39]. A drop in the CD56^bright^ and CD56^neg^ subsets of NK cells accounted for most of the observed decrease in peripheral blood NK cell numbers associated with seasonal (p = 0.0004 and p = 0.02) and 2009 pandemic H1N1 (p<0.0001 and p = 0.0001) influenza infections (Fig. 1C).

In contrast, in the study subjects receiving influenza vaccines, both absolute numbers and percentages of NK cells were significantly higher four days after immunization compared to one day after injection (p = 0.005 and p = 0.04, respectively). This observation could be attributed to an overall increase in all NK cells subpopulations, namely CD56^bright^ (p = 0.0001), CD56^dim^ (p = 0.007) and CD56^neg^ NK cells (p = 0.004) after an early drop in NK cell numbers one day after vaccination, particularly in the CD56^bright^ population (p = 0.008) (Fig. 1). Altogether, these data suggest that peripheral blood lymphocytes, including NK cells, home to infected tissues early in acute infection with both seasonal and 2009 pandemic H1N1 influenza strains, and that this recruitment might specifically involve CD56^bright^ NK cells. On the other hand, exposure to the inactivated virus through intramuscular route resulted in an initial drop ultimately leading to increased amounts of peripheral blood NK cells, which might be explained by NK cell proliferation and/or recruitment from the tissues.

### Different profiles of peripheral blood NK cell activation in seasonal or 2009 pandemic H1N1 influenza infection and influenza vaccination

**Figure 2:**
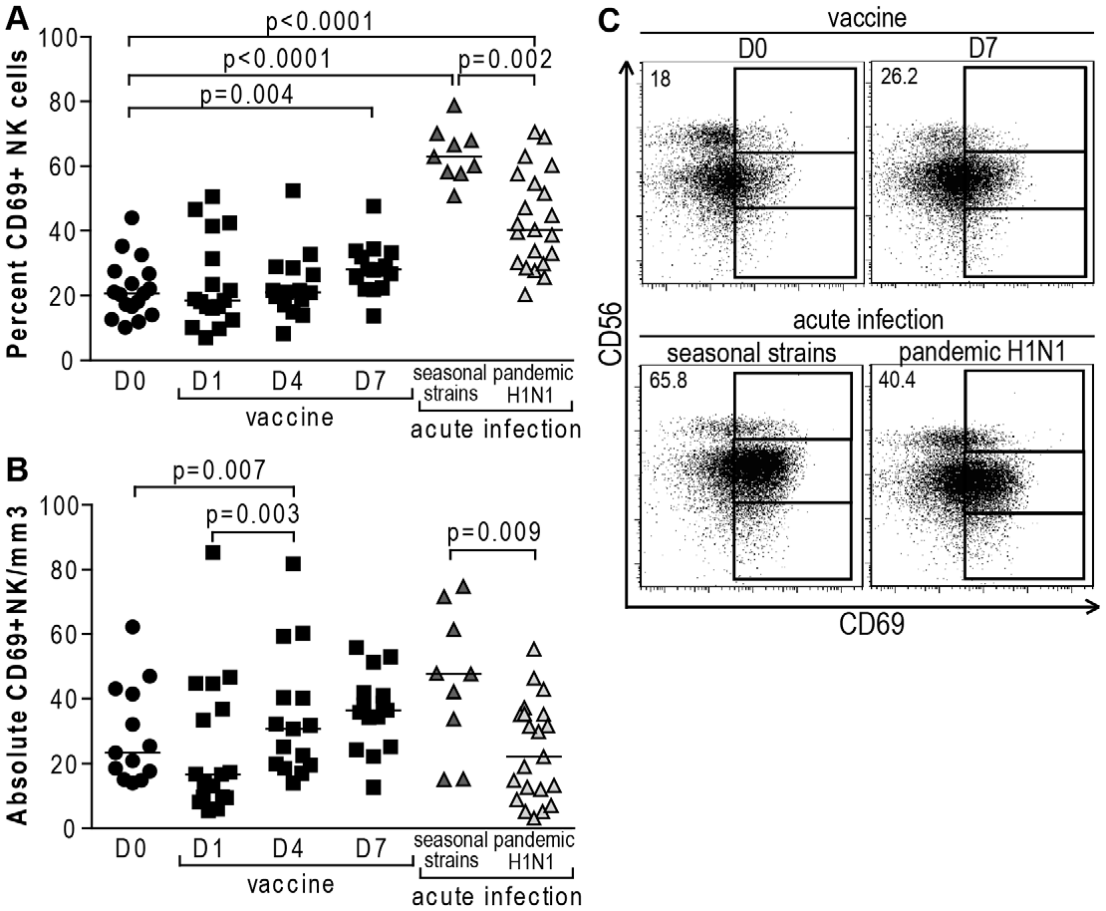
Changes in CD69 expression on NK cells in response to influenza infection and vaccination. Dot plots represent (A), percentages and (B), absolute numbers of CD69+ NK cells from 17 subjects prior to influenza vaccination, and at day 1, 4 and 7 post-vaccination, 9 patients with acute seasonal influenza and 21 with acute 2009 pandemic H1N1 influenza virus infection. Complete blood counts at baseline were only available for 13 of the vaccinated subjects. (•, healthy subjects prior vaccination; ▪, healthy subjects post-vaccination; dark grey ▴, acutely infected with 2009 seasonal influenza strains; light grey ▴, acutely infected with 2009 pandemic H1N1 influenza virus). (C) Representative primary flow panels show percentages of CD69+ CD56^bright^, CD56^dim^ and CD56^neg^ NK cells prior to and 7 days post-vaccination and in seasonal and pandemic influenza infection. Percentages of CD69+ total NK cells are indicated.

In order to further determine the NK cell response to influenza, we assessed the expression of the activation markers CD69 and CD25 (the α-chain of the IL-2 receptor) on NK cells from patients infected or vaccinated with influenza. Interestingly, influenza infection with seasonal strains was associated with significantly higher proportions and higher absolute numbers of CD69+ NK cells compared to swine influenza infection (p = 0.002 and p = 0.009, respectively) (Fig. 2). Disparities in age, fever and previous influenza vaccinations could not explain the observed differences in the NK cell activation status between individuals infected with 2009 pandemic H1N1 and seasonal influenza strains (data not shown). When compared to healthy individuals not exposed to influenza antigens, percentages but not absolute numbers of NK cells expressing CD69 were higher in acutely infected subjects (p<0.0001), suggesting that most of the peripheral blood NK cells that do not home to other tissues express CD69. CD69 expression also increased on NK cells from vaccinated individuals, with the highest proportions of activated NK cells observed one week following injection of the viral antigens (p = 0.004) (Fig. 2A).

**Figure 3:**
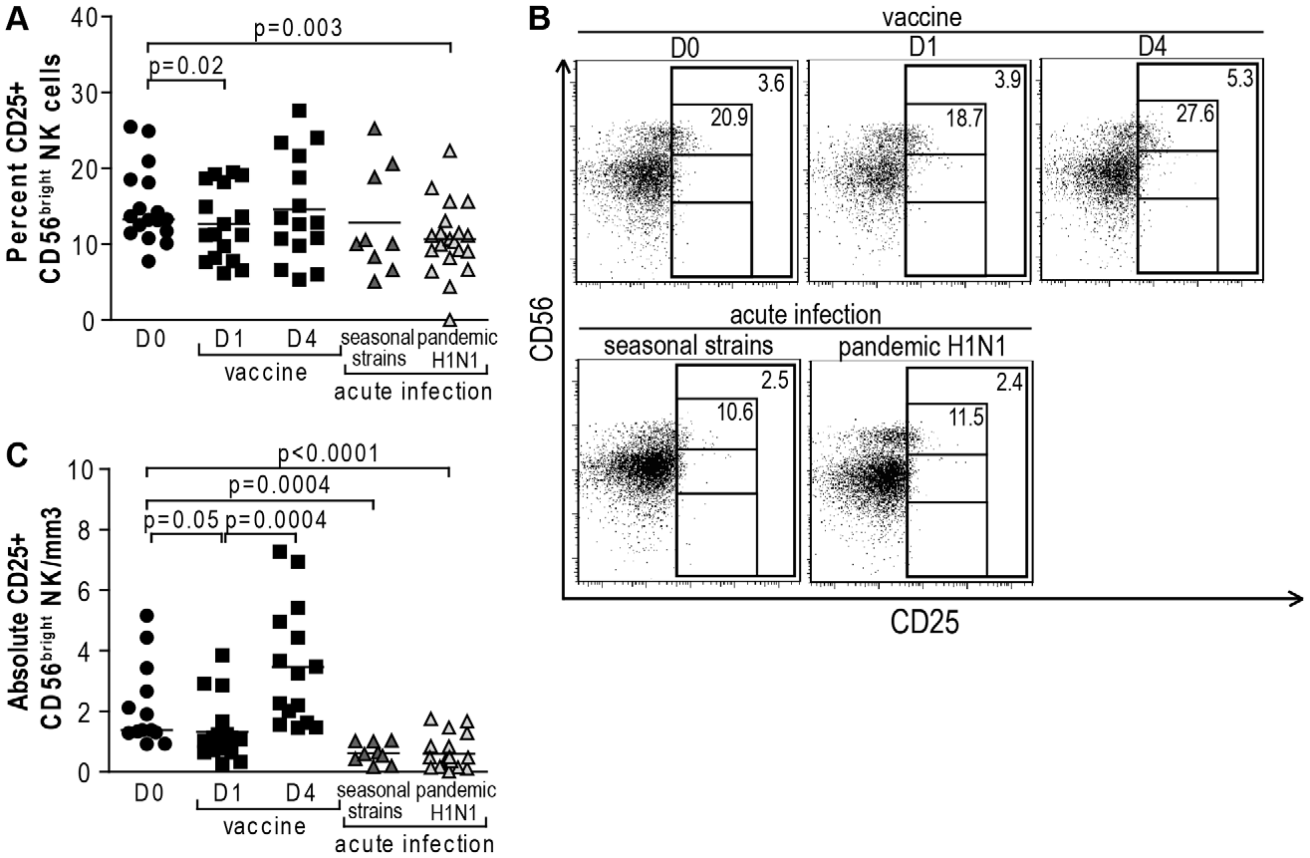
Changes in CD25 expression on NK cells in response to influenza infection and vaccination. (A) Dot plots represent percentages of CD25+ CD56^bright^ NK cells from 17 subjects prior to influenza vaccination, and at day 1 and 4 post-vaccination, 9 patients with acute seasonal influenza and 21 with acute 2009 H1N1 pandemic influenza. (B) Representative primary flow panels show percentages of CD25+ CD56^bright^, CD56^dim^ and CD56^neg^ NK cells prior to and at day 1 and 4 post-vaccination as well as in seasonal and pandemic influenza infection. Percentages of CD25+ total and CD25+ CD56^bright^ NK cells are indicated in the upper right corner of the total NK cell gate and CD56^bright^ NK cell gate, respectively. (C) Dot plots represent absolute numbers of CD25+ CD56^bright^ NK cells from 17 subjects prior to influenza vaccination, and at day 1 and 4 post-vaccination, 9 patients with acute seasonal influenza and 21 with acute 2009 pandemic H1N1 influenza virus infection. Complete blood counts at baseline were only available for 13 of the vaccinated subjects. (•, healthy subjects prior vaccination; ▪, healthy subjects post-vaccination; dark grey ▴, acutely infected with 2009 seasonal influenza strains; light grey ▴, acutely infected with 2009 pandemic H1N1 influenza virus). Horizontal lines indicate the median percentages. Differences where p<0.05 are indicated. D: day post-vaccination.

Furthermore, infection with the 2009 pandemic H1N1 influenza virus was associated with decreased percentages and absolute numbers of CD56^bright^ NK cells expressing CD25 compared to uninfected subjects (p = 0.003 and p<0.0001, respectively) (Fig. 3). An important drop in CD25+ NK cell counts also occurred in the CD56^bright^ CD25+ NK cell subset in patients with seasonal influenza infection (p = 0.0004) (Fig. 3C). Similar to CD56^bright^ NK cell numbers, influenza vaccination triggered an initial drop (p = 0.05) followed by an increase in CD56^bright^ CD25+ NK cells in the blood four days following administration of the vaccine (p = 0.0004) (Fig. 3C). CD25 is mostly expressed by CD56^bright^ NK cells, and therefore might explain why changes in CD25+ NK cells mirror those previously observed for the CD56^bright^ NK cell subset.

**Figure 4:**
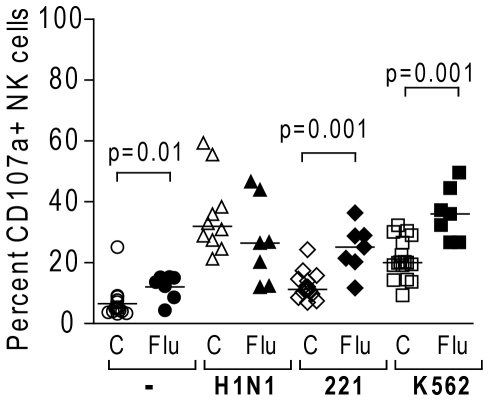
Functional response of NK cells from influenza-infected subjects. Dot plots represent percentages of CD107a+ NK cells from 17 healthy subjects prior to influenza vaccination (C) and 7 subjects with acute seasonal influenza infection (Flu) following incubation with medium alone, H1N1 PR8 influenza virus for 16 h, or 221 and K562 target cells for 4 h at an effector∶ target ratio of 10∶1. Influenza-stimulation of NK cells was only performed on 10 subjects prior to vaccination (C).

In accordance with the phenotypic data, peripheral blood NK cells from patients with seasonal influenza displayed higher levels of CD107a surface expression in response to MHC-devoid 221 and K562 cell lines compared to NK cells from control subjects (p = 0.001) (Fig. 4). In contrast, NK cells that were exposed to seasonal influenza virus *in vivo* tended to have a decreased response to the human H1N1 influenza virus *in vitro* (Fig. 4). IFN-γ production following NK cell stimulation with target cells or influenza was generally low and did not differ between healthy and infected subjects (median percentages with ranges after subtracting background in healthy vs. influenza-infected following stimulation with H1N1: 0.2 (0–4.3) vs. 0.7 (0.2–1.9); 221 cells: 0.4 (0–5.2) vs. 1.3 (0–2.6); K562 cells: 0.7 (0–9.8) vs. 2.8 (1.1–5.1); data not shown). CD107a upregulation or IFN-γ production in response to 221 or K562 cells did not vary over time in vaccinated individuals (data not shown).

These results indicate that infection with 2009 pandemic H1N1 influenza virus lead to fewer activated NK cells in the blood of infected subjects than did infection with seasonal influenza viruses and that increased numbers of activated NK cells, and notably CD56^bright^ NK cells, are found in the peripheral blood between 4 to 7 days after intramuscular vaccination.

### Infection with seasonal and 2009 pandemic H1N1 influenza viruses leads to similar changes in cytokine plasma levels

**Figure 5:**
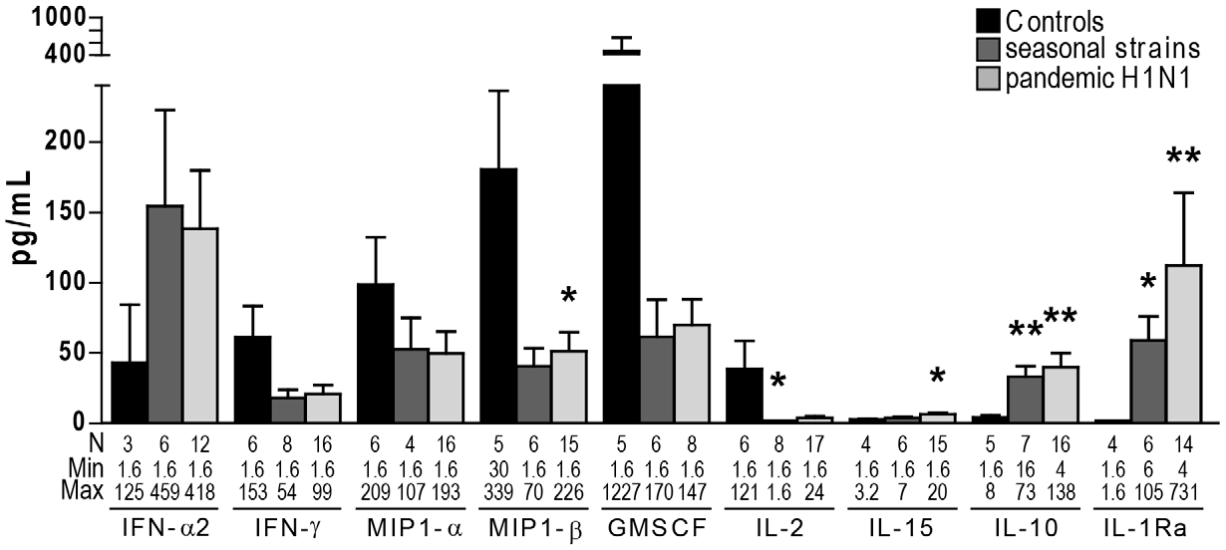
Changes in plasma cytokine concentrations associated with acute influenza infection. Columns represent mean concentrations of indicated cytokines in 7 controls (black), 9 patients with acute seasonal influenza (dark grey) and 21 with acute 2009 pandemic H1N1 influenza (light grey). For each column, N represents the number of values included in the analysis based on a coefficient of variation <30. Low out of range values were set to half of the lowest standard value (1.6 pg/mL). Bars indicate standard error of the mean. * represents *p*<0.05; ** represents *p*<0.001 and *** represents *p*<0.0001.

Decreased amounts of peripheral blood activated CD56^bright^ NK cells in acute influenza patients may reflect that the function of these particular cells is recruited to infected tissues and/or that this subset of NK cells is subjected to increased cell death rates. As CD56^bright^ NK cells have immunoregulatory properties [38], we examined plasma levels of a panel of cytokines, including those that can be produced by NK cells, such as IFN-γ, MIP-1α and β, GM-CSF and IL-10, and those that can induce NK cell activation, proliferation and differentiation, such as IFN-α, IL-2, and IL-15 [40], [41]. Plasma concentrations of IFN-γ, MIP-1α, MIP-1β and GM-CSF tended to be lower in patients infected with seasonal and 2009 pandemic H1N1 influenza viruses than in healthy individuals, yet a significant decrease was only observed for MIP-1β in subjects with pandemic H1N1 influenza infection (p = 0.03) (Fig. 5). In contrast, we found markedly increased levels of cytokines with anti-inflammatory properties including IL-10 (p = 0.005 and 0.002) and IL-1Ra (p = 0.01 and p = 0.003) in influenza-infected patients. Regarding cytokines involved in NK cell activation, plasma levels of IL-2 decreased in infected patients, notably in seasonal influenza infection (p = 0.04), while the concentration of IL-15 was slightly higher in acute influenza, and particularly in subjects with 2009 pandemic H1N1 influenza infection (p = 0.04). Acute influenza was also associated with an overall three-fold increase in plasma IFN-α2 (not significant). Importantly, there were no remarkable differences in cytokine concentrations between patients infected with 2009 pandemic H1N1 influenza virus and those infected with seasonal influenza strains. Cytokines concentrations were very low and stable over time in the plasma of vaccinated subjects (data not shown). In summary, low levels of cytokines produced by NK cells, among other cell types, and significantly increased amounts of anti-inflammatory cytokines were observed in the blood of subjects with acute influenza infection.

## Discussion

In this study, we investigated changes in peripheral blood NK cell numbers and activation in response to acute influenza infection, comparing infection with 2009 influenza circulating strains and 2009 pandemic H1N1 influenza virus. NK cell phenotype and function following intramuscular immunization using trivalent inactivated influenza virus vaccines from 2008–2009 or 2009–2010 were also examined. Influenza infection and vaccination both affected NK cell counts, the former leading to decreased and the latter to increased numbers of peripheral NK cells, with the most significant changes affecting CD56^bright^ NK cells expressing the marker of immune activation CD25. Exposure to influenza antigens was associated with increased percentages of CD69+ NK cells, notably in the peripheral blood of patients infected with seasonal strains, consistent with a virus-dependent state of NK cell immune activation and a more potent cytotoxic function against target cell lines than NK cells from healthy subjects. Compared to healthy unvaccinated individuals, acute influenza infection triggered significant variations in cytokine concentrations, and a similar cytokine profile was observed in acutely infected patients independently of the infecting strain.

Reduced numbers of peripheral blood NK cells, and particularly activated CD56^bright^ NK cells, in patients with acute influenza infection might reflect homing of these cells to other tissues, including those of the respiratory tract, as previously reported in subjects infected with an H1N1 influenza strain [16]. Alternatively, increased apoptosis and activation-induced cell death might explain the observed decrease in circulating NK cells. Apoptosis has been proposed to participate in the severe depletion of peripheral blood lymphocytes associated with other viral infections [42], [43]. In accordance with this hypothesis, lung inflammatory infiltrates from one subject with fatal H1N1 influenza infection were devoid of NK cells [13]. Moreover, it has been recently shown that influenza can infect NK cells, thereby triggering NK cell death, and potentially providing a mechanism for the virus to escape innate immune defenses [18], [44]. Further analysis will be required to evaluate the role played by trafficking to infected tissues and/or immune cell death in influenza-associated lymphopenia.

Changes in NK cell counts observed following vaccination suggest an initial recruitment to the tissues, and potentially at the site of injection, followed by a substantial release of NK cells, and notably CD56^bright^ NK cells, in the blood. NK cell proportions were not altered in another longitudinal study examining NK cell responses in individuals immunized against influenza. However, frequencies of NK cells were first monitored one week after vaccination, at a time when NK cell numbers also did not significantly differ compared to baseline in the samples we analyzed, explaining the discrepancy between our findings, and suggesting that changes in NK cells following vaccination occur rapidly [45].

CD56^bright^ NK cells have a higher capacity to produce cytokines as well as a more important proliferative potential than other NK cell subsets [46]. Therefore, NK cell proliferation and production of inflammatory cytokines rather than cytolytic functions might be required to promote effective innate immune responses and control influenza infection. While we are not able to investigate whether NK cells do proliferate in the respiratory tract of infected individuals, increased numbers of peripheral blood activated NK cells, and notably CD56^bright^ NK cells, four days after vaccination might indicate expansion and/or recruitment of NK cells from the tissues to the blood.

In order to further assess NK cell activation in influenza infection and vaccination, we looked at the expression of CD69 and CD25 at the surface of NK cells and quantified NK cell function in response to H1N1 or 221 and K562 target cells *ex vivo*. Acute influenza infection was associated with increased percentages of CD69+ and decreased absolute numbers of CD25+ NK cells, suggesting that CD25+ NK cells, and therefore activated CD56^bright^NK cells, might be recruited to the site of infection and/or might undergo increased apoptosis rates, while CD69 is a less specific marker of activation that is upregulated on all NK cells subsets. Increased CD69 expression conferred a higher NK cell-mediated cytotoxic function against K562 or 221 cell lines yet a diminished degranulatory activity in response to a secondary stimulation with H1N1, supporting the hypothesis that NK cells that can efficiently respond to influenza have been recruited to infected tissues and are not circulating in the periphery any more. Alternatively, NK cells might have become anergic to further restimulation. For instance, exposure to influenza antigens leads to the downregulation of NKp46, an activating NK cell receptor that can recognize influenza hemagglutinin, an observation that might explain the decreased NK cell response to influenza re-stimulation [47].

Interestingly, percentages of peripheral blood CD69+ NK cells significantly differ between patients infected with seasonal influenza strains and subjects with acute swine influenza infection. While we did not find any correlation with fever, lower proportions of NK cells expressing CD69 in swine influenza infection might be associated with generally more serious symptoms, in accordance with a recent study describing extremely low numbers of NK cells in cases of severe and/or lethal 2009 pandemic H1N1 influenza infection, and suggesting that NK cell proportions correlate with the severity of the diseases [13]. Further investigations, such as gene expression studies, will be required to identify potential factors accounting for the observed differences in NK cell activation in seasonal and 2009 pandemic H1N1 influenza virus infections.

Our results support a role for NK cells, and particularly for CD56^bright^ NK cells, in the response to influenza. As the CD56^bright^ NK cell subset is endowed with immunoregulatory properties, and the pathogenicity of virulent influenza strains has been associated with altered innate responses and hypercytokinemia [48]–[50], we quantified the levels of various cytokines in the plasma of infected and vaccinated subjects. Decreased concentrations of several pro-inflammatory cytokines, including IL-2 and IFN-γ, of the growth factor GM-CSF and of the chemokines MIP-1α and MIP-1β were observed in infected individuals compared to healthy subjects, possibly reflecting an uptake of these cytokines by other immune cells. Alternatively, these cytokines might be secreted locally in other tissues than blood by cells that have been recruited to the site of infection. However, it is very likely that massive local production of cytokines would lead to spillover into the systemic circulation. Elevated plasma levels of IL-6, IL-12 and IFN-γ in patients with severe influenza were recently reported [51]. Our study subjects were enrolled through the Massachusetts General Hospital emergency room and therefore presented symptoms, yet the fact that they had a moderate rather than a severe disease might explain the discrepancy between our results and those published by Heltzer et al. Also, samples from acutely infected individuals might have been collected too late after infection to identify IL-12 and IFN-γ in the plasma. IL-6 plasma levels were not examined in our study. Nevertheless, timing issues could not explain the suppression of cytokines observed in our influenza-infected subjects. In addition, elevated concentrations of several pro-inflammatory cytokines have been detected in the acute symptomatic phase of many viral infections such as severe acute respiratory syndrome virus, human immunodeficiency virus, hepatitis C virus and severe influenza virus infections [48], [52]–[54]. One hypothesis is that low NK cell numbers due to significant NK cell death might account for the decreased NK cell production of cytokines, including IFN-γ, GM-CSF, MIP-1α and MIP-1β, detected in our patients. Surely, further experiments are warranted to understand the mechanisms underlying the low pro-inflammatory cytokine levels observed in our patients. Nonetheless, in accordance with previously published studies, we report increased levels of IFN-α2 in the plasma of influenza-inoculated subjects [55], yet this cytokine is more likely to derive from plasmacytoid dendritic cells than from NK cells. Changes in IL-2 and IL-15 levels are consistent with a key role for these cytokines in the NK cell response to influenza infection as we and others have reported that IL-2 is required to enhance IFN-γ production by NK cells, while IL-15 promotes NK cell proliferation in response to influenza (data not shown and [56]).

In contrast, levels of two anti-inflammatory cytokines, IL-1ra and IL-10, were elevated in influenza infection, probably to limit damages that could be provoked by an over-activated immune system. However, the role played by these cytokines in influenza infection is not clear yet. While IL-1ra treatment has been shown to increase the survival rate of influenza-infected mice [57], the impact of IL-10 on the outcome of influenza infection in mouse models is still controversial [58]–[60]. Further investigations to better characterize the role played by these cytokines in seasonal influenza infection, and notably to identify IL-10- and IL-1ra-secreting immune cell subsets, are warranted, as other regulatory responses, such as T cell responses, probably contribute to the observed immune suppressive profile.

Overall, influenza exposure was associated with changes in peripheral blood NK cell numbers and activation, consistent with a role for NK cells in the innate immune response to influenza antigens. These data contribute to a better understanding of the innate immune mechanisms at play during acute influenza infection and vaccination in humans, providing a basis for further analysis into the contribution of these cells in the control of human influenza infection.

## Materials and Methods

### Ethics statement

The study was approved by the Massachusetts General Hospital Institutional Review Board and each subject gave written informed consent for participation in the study.

### Study subjects

Peripheral blood mononuclear cells (PBMCs) were isolated from blood samples collected at day 0, 1, 4 and 7 from 17 healthy volunteers (11 women and 6 men; median age 27 years, range 22–57 years) immunized intramuscularly with 0.5 ml inactivated influenza virus vaccines containing 15 ug purified HA from each of the three seasonal influenza strains. 13 subjects were vaccinated with Fluarix® 2008–2009 formula (GlaxoSmithKline, Dresden, Germany) containing the following inactivated virus strains: A/Brisbane/59/2007 IVR-148 (H1N1), A/Uruguay/716/2007 NYMC X-175C (H3N2) and B/Brisbane/3/2007 (influenza B virus) and 4 subjects received Fluzone® 2009–2010 formula (Sanofi Pasteur, Lyon, France) which is a mixture of inactivated A/Brisbane/59/2007 IVR-148 (H1N1), A/Uruguay/716/2007 NYMC X-175C (H3N2) and B/Brisbane/60/2008 (influenza B virus) influenza viruses. To study NK cell responses to influenza infection, 30 individuals presenting to the Massachusetts General Hospital emergency room with acute influenza symptoms were enrolled in a second cohort (14 women and 16 men; median age 32 years, range 21–62 years). Nine patients (3 women and 6 men; median age 35 years, range 21–46 years) were enrolled in January and February 2009 after being positively diagnosed for influenza with a rapid test and were probably infected with influenza A, which was the most common strain in circulation at that time. In addition, 21 patients (11 women and 10 men; median age 32 years, range 21–62 years) clinically diagnosed with pandemic H1N1 influenza A virus infection between October and December 2009 participated in the study. Blood was drawn only at the time of enrollment. For both cohorts, a complete blood count and a white blood cell differential count were performed at each blood draw.

### Analysis of NK cells surface receptors expression and NK cell function by flow cytometry

PBMCs were isolated by Histopaque density gradient centrifugation (Sigma). To monitor changes in NK cells receptor expression, thawed PBMCs were stained using CD56-A700, CD16-APC-Cy7, CD3-Pac Blue and either CD69-PE-Cy7 or CD25-PE-Cy7 (BD Biosciences). The LIVE/DEAD® Fixable Blue Dead Cell Stain Kit was used prior surface staining to exclude dead cells. NK cell function was quantified after stimulation of fresh PBMCs either with major histocompatibility complex class I (MHC-I)–devoid K562 and 221 cell lines (ATCC) at an effector-to-target cell ratio of 10∶1 as previously described [61] or with the A/PR/8/34 H1N1 influenza virus (Charles River Laboratories, Wilmington, Massachusetts, USA). Influenza infection was performed by adding the virus at an MOI of 5.2 to 10^6^ cells resuspended in 0.1 ml of RPMI-1640 medium without serum. After one hour of incubation at 37 C with 5% CO2, RPMI-1640 supplemented with 10% fetal bovine serum, 2 mM L-glutamine, 100 µg/ml streptomycin and 100 U/ml penicillin was added to a final volume of 1 ml. 7 µL/ml CD107a-PE-Cy5 antibody (BD Biosciences) and monensin (GolgiStop; BD Biosciences) at a final concentrations of 0.9 nM were added immediately to all reaction tubes and the total stimulation lasted for 4 (cell lines) or 16 (influenza) hours. Unstimulated PBMCs were similarly treated in parallel to define the background level of degranulation and PMA/ionomycin (2.5 and 0.5 ug/ml, respectively) served as the positive control. Fixed cells were analyzed on an LSRII system using FacsDiva version 8.8.3 (BD Biosciences). The frequency and phenotypes of NK cells were defined using FlowJo version 7.5.5 (Treestar).

### Quantification of plasma cytokines

Plasma concentrations of cytokines in influenza vaccinated and infected patients were determined using a Milliplex Human Cytokine Multiplex Immunoassay Kit (Millipore) and read on a Bio-Plex 200 reader (Bio-Rad Laboratories). Values for which the coefficient of variation (%CV) was above 30 were excluded (ranging from 1 to 13 exclusions), resulting in N values analyzed for each cytokine and each population, as indicated in figure 5. Low out of range values with a %CV below 30 were set to half of the lowest standard value (1.6 pg/mL), while low out of range values that were extrapolated beyond standard range by the software were set at the value of the lowest standard (3.2 pg/mL). The kit was customized to detect GM-CSF, IFN-α2, IFN-γ, IL-1ra, IL-1α, IL-1β, IL-2, IL-10, IL-12(p40), IL-12(p70), IL-15, IL-17, MIP-1α, MIP-1β and TNF-α. Plasma cytokine levels from subjects prior to vaccination were used as control.

### Statistical analyses

Statistical analyses were performed using the GraphPad Prism software version 5.04. The non-parametric Wilcoxon signed-rank test was used to assess longitudinal differences in phenotype frequencies in vaccinated individuals. The non-parametric Mann-Whitney test was used to compare phenotype frequencies and cytokine concentrations between influenza-infected patients and subjects prior vaccination. P-values of <0.05 were considered significant.
